# Distinct expression profiles of regulatory RNAs in the response to biocides in *Staphylococcus aureus* and *Enterococcus faecium*

**DOI:** 10.1038/s41598-021-86376-y

**Published:** 2021-03-25

**Authors:** Loren Dejoies, Killian Le Neindre, Sophie Reissier, Brice Felden, Vincent Cattoir

**Affiliations:** 1 Department of Clinical Microbiology, Rennes University Hospital, Rennes, France; 2 Inserm UMR_S 1230, Bacterial Regulatory RNAs and Medicine, University of Rennes 1, Rennes, France; 3 National Reference Center for Antimicrobial Resistance (Lab ‘Enterococci’), Rennes, France

**Keywords:** Bacterial genetics, Small RNAs, Antimicrobial resistance

## Abstract

The aim of the study was to characterize the antimicrobial activity of clinically-relevant biocides (chlorhexidine digluconate, benzalkonium chloride, PVP-iodine and triclosan) and to determine the sRNA expression profiles under biocide exposure in two major bacterial opportunistic pathogens, *Enterococcus faecium* and *Staphylococcus aureus*. In vitro activities were evaluated against *S. aureus* HG003 and *E. faecium* Aus0004. We determined MIC, MBC, sub-inhibitory concentrations (SIC) and growth curves under SIC conditions. sRNA expression study under SIC exposure of biocides was performed by RT-qPCR on 3 sRNAs expressed in *S. aureus* (RNAIII, SprD and SprX) and the first 9 sRNAs identified as expressed in *E. faecium*. MICs were higher against *E. faecium* than for *S. aureus*. Growth curves under increasing biocide concentrations highlighted two types of bactericidal activity: “on/off” effect for chlorhexidine, benzalkonium chloride, PVP-iodine and a “concentration-dependent” activity for triclosan. Exposure to biocide SICs led to an alteration of several sRNA expression profiles, mostly repressed. The distinct biocide activity profiles must be evaluated with other compounds and bacterial species to enrich the prediction of resistance risks associated with biocide usage. Biocide exposure induces various sRNA-mediated responses in both *S. aureus* and *E. faecium*, and further investigations are needed to decipher sRNA-driven regulatory networks.

## Introduction

Resistance to antibiotics keeps increasing worldwide and can lead to treatment failures of severe bacterial infections. “ESKAPE” pathogens include healthcare-relevant Gram-positive and Gram-negative bacteria displaying high capacities for acquisition of resistance determinants, and causing numerous multi-drug resistant (MDR) infections worldwide^1^. *Staphylococcus aureus* and *Enterococcus faecium* are two Gram-positive bacteria represented in this ESKAPE group and stand as major human nosocomial pathogens^2^. Searching for novel antimicrobial strategies is essential, but must not be the only option to struggle against infections. Indeed, limiting their occurrence, notably hospital-acquired infections can be achievable with appliance of rigorous hygiene practices in healthcare units. To fulfill these objectives, biocides (incl. antiseptics, disinfectants) are massively used at the hospitals^3,4^. They also include preservative agents and are therefore widely used in industries (e.g. food, cosmetics). As a consequence, the bioaccumulation of various biocides in the environment (food, water, soils) has been evidenced^5^, and some of them, such as triclosan, have been detected in human urine and breast milk^6,7^. Nevertheless, the widespread use of biocides based on clinically-evidenced antimicrobial efficacy is in contrast with the lack of knowledge about their molecular mechanisms of action and resistance. Some are predicted to share common targets with antibiotics, such as cell membranes (chlorhexidine, benzalkonium chloride), while halogen-based biocides (PVP-iodine, triclosan) could have a global antimicrobial effect by inducing an oxidative stress in bacterial cell after passive diffusion through cell membrane^8,9^. In a context of global health concern, the exacerbation of emerging antimicrobial resistance after overuse and/or misuse of biocides has been hypothesized^10^. Indeed, evidences for a link between heavy usage of biocides and emergence of antibiotic resistance are currently highlighted, even though the molecular mechanisms are partially elucidated^11–16^. Furthermore, official guidelines in biocide susceptibility testing are still pending, despite recent propositions have led significant advances in the standardization of procedures^17,18^. As a consequence, the high variability of published results related to different procedures contributes to the misunderstanding of cross-/co-resistance mechanisms. Among various strategies triggered by bacteria to cope with environmental changes, regulatory RNAs (sRNAs) are key players among intertwined regulatory networks that mediate dynamic adaptations by modulating gene expression^19,20^. Bacterial adaptation mediated by sRNAs has been exclusively studied under antibiotic conditions^21,22^, but we presume that a biocide stress, especially at subinhibitory concentrations (SICs, concentrations below the MIC, which do not affect bacterial growth), could trigger sRNA-regulated metabolic pathways as well. Among Gram-positive human pathogens, sRNAs have been studied in *S. aureus,* with an estimated number close to 50 *bona fide* sRNAs^23^. Among them, three can be qualified as “clinically-relevant”: RNAIII, implicated into the quorum sensing, allowing the transition between expression of adhesive and colonization factors^24^, SprX that contributes to glycopeptide resistance by repressing stage V sporulation protein (spoVG) expression^25^, and SprD that regulates expression of an immune evasion protein (Sbi) and is required for infection on animal models^26^. In *Enterococcus* spp., sRNAs have been first described in *Enterococcus faecalis*^27,28^*.* In *E. faecium*, the first nine sRNAs whose biological functions remain unknown have been recently discovered in our lab^29^.

In this study, we aimed to characterize the antimicrobial activity of four clinically-relevant biocides (chlorhexidine digluconate, benzalkonium chloride, PVP-iodine and triclosan), and to determine the sRNA expression profiles under biocide stress in two major ESKAPE Gram-positive pathogens, *E. faecium* and *S. aureus*. For this aim, we developed different approaches to assess the behavior of those bacteria under biocidal exposure.

## Results

### Biocide susceptibility testing

MICs of chlorhexidine digluconate and benzalkonium chloride were two-fold higher against *E. faecium* than *S. aureus* (4 mg/L and 2 mg/L, respectively), while MICs of triclosan were four-fold higher (8 mg/L and 2 mg/L, respectively) (Table 1)*.* By contrast, MICs of PVP-iodine were identical for both species and 1000-fold higher than the other biocides (2048 mg/L). MBC/MIC ratios of the four tested biocides suggested a bactericidal activity against both *E. faecium* and *S. aureus* (ratio ≤ 4) (Table 1)^9^.

**Table 1:** Antimicrobial activities of chlorhexidine digluconate, benzalkonium chloride, triclosan and PVP-iodine on *S. aureus* HG003 and *E. faecium* Aus0004.

	*S. aureus* HG003				*E. faecium* Aus0004			
	CA-MHB medium		BHI medium		CA-MHB medium		BHI medium	
	MIC_48h_ (mg/L)	MBC_24h_ (mg/L)	MBC/MIC ratio	SIC (mg/L)	MIC_48h_ (mg/L)	MBC_24h_ (mg/L)	MBC/MIC ratio	SIC (mg/L)
Chlorhexidine digluconate	2	4	2	1	4	4	1	0.5
Benzalkonium chloride	2	8	4	1	4	8	2	2
Triclosan	2	8	4	0.016	8	16	2	0.25
PVP-iodine	2048	2048	1	1024	2048	2048	1	1024

CA-MHB: cation-adjusted Mueller–Hinton broth; BHI: Brain Heart Infusion; MIC_48h_: minimal inhibitory concentrations after 48 h of incubation; MBC_24h_: minimal bactericidal concentrations after 24 h of incubation; SIC: sub-inhibitory concentration.

Bacterial growth under gradient concentrations of biocide revealed that SIC of benzalkonium chloride and triclosan were higher for *E. faecium* than for *S. aureus,* whereas SIC of chlorhexidine digluconate was higher for *S. aureus* than for *E. faecium*. PVP-iodine SICs were identical for both bacteria and were again about 1000 times higher than the three other biocides (Table 1).

### Antibacterial activity profiles

**Figure 1:**
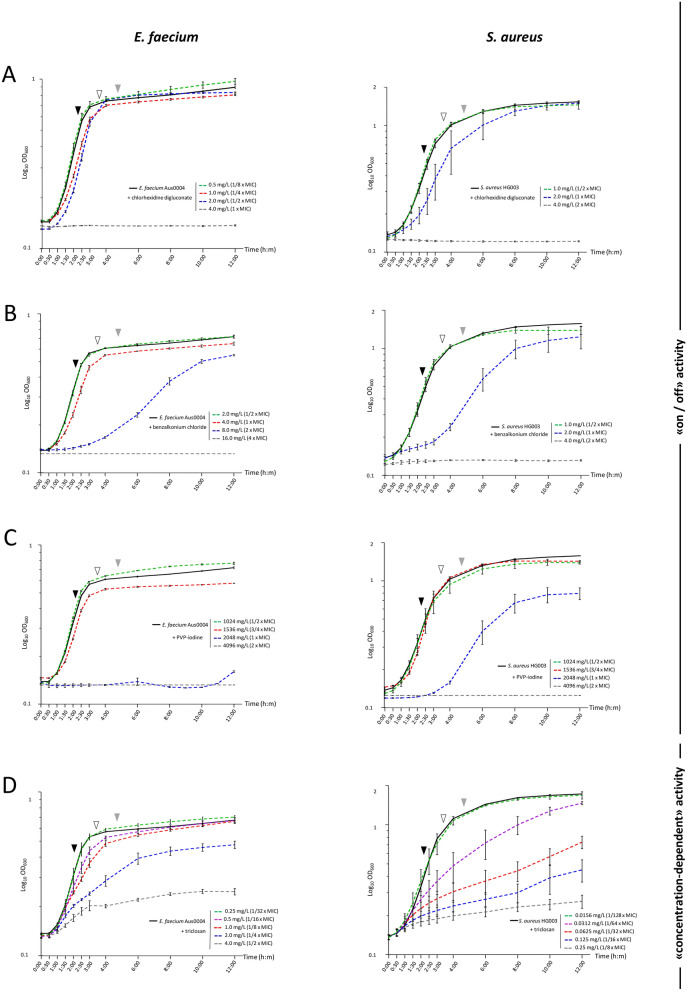
Growth kinetics of *E. faecium* Aus0004 (lefts panels) and *S. aureus* HG003 (right panels) under gradients of chlorhexidine (range from 0.5 to 4.0 mg/L) (**A**), benzalkonium chloride (range from 1.0 to 16.0 mg/L) (**B**), PVP-iodine (range from 1024 to 4096 mg/L) (**C**) and triclosan (range from 0.0156 to 4 mg/L) (**D**). Growth curves under sub-inhibitory concentrations (SICs), defined as the highest concentration of biocide for which the standard deviation of the growth curve overlapped with the one of the biocide-free control, are represented in green dashed line. Growth stages considered for sRNA expression corresponded to mid (ME, full black triangle), late (LE, empty black triangle) exponential growth and early stationary (ES, full grey triangle) phases. Error bars indicate standard deviations from three independent experiments.

SICs of chlorhexidine digluconate, benzalkonium chloride and PVP-iodine were reached at 1/2× MIC for *S. aureus*, as well as for *E. faecium* except with chlorhexidine digluconate (SIC at 1/8× MIC). SICs of triclosan were much lower than MICs at 1/32× and 1/128× MIC for *E. faecium* and *S. aureus*, respectively (Fig. 1 and Table 1). This difference between chlorhexidine, benzalkonium chloride and PVP-iodine in one side, and for triclosan on another was also detected after the analysis of the growth curves. Indeed, two profiles of antibacterial activity were highlighted: chlorhexidine digluconate, benzalkonium chloride and PVP-iodine revealed a weak concentration-dependant activity illustrated by an “on/off” effect on bacterial growth between 1 two-fold dilution. Triclosan revealed a strong concentration-dependent activity resulting in the gradual inhibition of bacterial growth while biocide concentrations increased (Fig. 1).

### Overall repression of *E. faecium* sRNAs expression under biocide exposure

**Figure 2:**
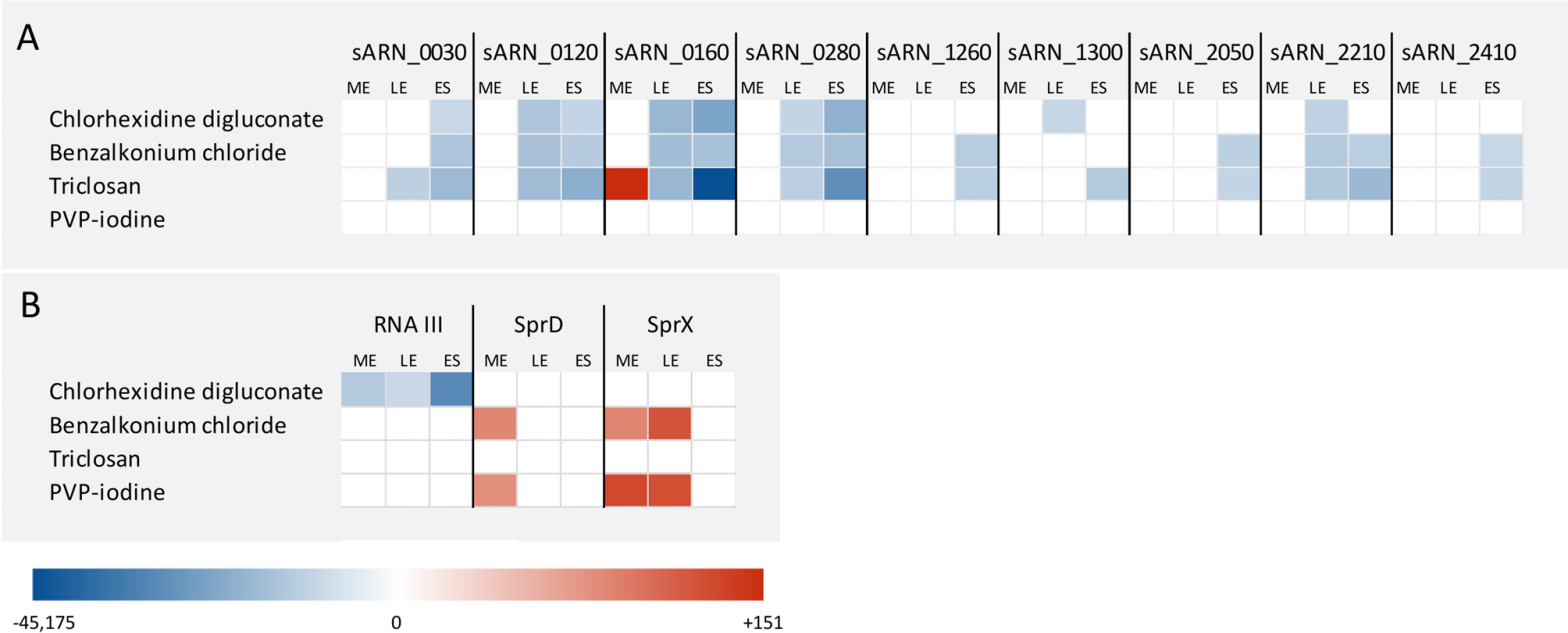
Heat maps illustrating variations in sRNA expression of *E. faecium* Aus0004 (**A**) and *S. aureus* HG003 (**B**) at mid exponential, late exponential and early stationary growth phases after exposure to SICs of chlorhexidine digluconate, benzalkonium chloride, PVP-iodine and triclosan. RNA counts were normalized against house-keeping genes (*tmRNA* for *S. aureus* and *adk* for *E. faecium*) using the comparative cycle threshold ΔΔCt method and are expressed in Log_2_ fold change. Non-significant shifts in sRNA expression appear in white, significant induction (*P* < 0.05) appears in red, significant repression (*P* < 0.05) are in blue.

Expression profiles of sRNA under each biocide condition are presented in Fig. 2A and Table S1. No significant shifts at the transcript level were observed at the ME phase, except for sRNA_0160 under triclosan (+ 151; *P* = 0.0008). During the later stages, a global repressive trend of sRNAs steady state expression levels was observed. At the LE phase, their expression profiles were similar under chlorhexidine, benzalkonium chloride and triclosan exposure, with a significant decrease of expression for four sRNAs (sRNA_0120, sRNA_0160, sRNA_0280 and sRNA_2210). Besides, sRNA_1300 expression was repressed only under chlorhexidine, while sRNA_0030 expression was repressed only under triclosan. At the ES phase, the expression of some sRNAs was significantly decreased under chlorhexidine (4 out of 9) and benzalkonium chloride (8 out of 9), and the expression of all 9 sRNAs was switched off under triclosan, notably with sRNA_0160 and sRNA_0280 practically extinct (− 45,175; *P* < 0.0001 and − 1009; *P* = 0.0002 respectively). Interestingly, no significant shifts were observed under PVP-iodine at none of the three growth stages.

### Impacts of biocide exposure onto *S. aureus* sRNAs expression

sRNA expression profiles under each biocide condition are presented on Fig. 2B and Table S2. RNAIII expression profile was significantly influenced (*P* < 0.05) only under chlorhexidine digluconate SIC. Its expression was slightly repressed at ME and LE stages (− 26.8; *P* = 0.04 and − 10.6; *P* = 0.0008 respectively), and mostly at the ES phase (− 1310; *P* = 0.04). SprX expression profile was altered under two biocides. We observed an increase of expression at the ME and LE stages, then return to normalcy at the DS stage under SICs of benzalkonium chloride (+ 18.6; *P* = 0.02, + 57.9; *P* = 0.04, + 1.6; *P* > 0.05) and PVP-iodine (+ 77.6; *P* = 0.02, + 66.2; *P* = 0.001, + 4.9; *P* > 0.05). SprD expression showed significant shifts under SICs of benzalkonium chloride (+ 18.2; *P* = 0.003) and PVP-iodine (+ 14.7; *P* = 0.003) only at the ME stage. A triclosan exposure did not lead to significant changes in the expression profiles of RNAIII, SprX and SprD.

## Discussion

As expected, the four biocides tested here possessed a potent antimicrobial efficacy and revealed a bactericidal action against both *E. faecium* and *S. aureus* strains (MBC/MIC ratios ≤ 4)^30^. The antimicrobial activity of high biocide concentrations found in commercial products (at least at 100-times the MICs) was not investigated in this study because we assessed the effects of low concentrations, mimicking those detected in the environment after their usual uses and misuses. The higher MIC values of chlorhexidine gluconate, benzalkonium chloride and triclosan measured for *E. faecium* suggest a reduced susceptibility to biocides as compared to *S. aureus*. These results are consistent with inherent ability of *Enterococcus* spp. to cope with antimicrobial stress^31^, even if they are unlikely exposed to biocides as part of the gastro-intestinal flora, by contrast with *S. aureus* and its cutaneous tropism. These results showing higher MICs in *Enterococcus* spp are consistent with other studies^18,32^. MIC values are in similar ranges and about 2- to 4-times higher in *E. faecium* than in *S. aureus* for every biocide, except for triclosan for which MIC value in *S. aureus* determined in our study is 16- to 30-times higher than in other studies^18,32^. This discrepancy reveals how raw values can be hardly comparable from one study to another due to a large variability in protocols and interpretation criteria, in addition to intrinsic differences between the tested strains. Indeed, the detection of non-susceptible isolates can fluctuate depending on methodologies used for data acquisition and interpretation. Also, standard protocols to assess the impacts of biocides on bacterial resistance onset are lacking. These major methodological issues were pointed out in 2009 by the Scientific Committee on Emerging and Newly Identified Health Risks (SCENIHR) which recommended standardized methodologies and international surveillance programs to monitor the emergence of biocide resistance (https://ec.europa.eu/health/ph_risk/committees/04_scenihr/docs/scenihr_o_021.pdf). From there, increasing scientific investigations have led to practical propositions about standardization of biocide susceptibility testing and epidemiological cut-off assessments^17,18^.

Regretfully, we were unable to analyse the MIC/MBC and the SIC data altogether because the culture media were different between the experiments. Indeed, BHI medium was used to perform the biocide stress characterization experiment under sub-lethal biocide concentration because no exploitable data were collected in CA-MHB medium.

Based on the analysis of growth curves under sub-lethal conditions, we enlightened two distinct profiles of antimicrobial activity, independently from their bacterial targets. Chlorhexidine digluconate and benzalkonium chloride, both responsible for bacterial membranes disruption^8,9^, as well as PVP-iodine inducing an oxidative stress^8,9^, displayed a weak concentration-dependent effect mimicking an “on–off” effect. These results strengthen the need of concentrated commercial products in clinical use to ensure an optimal antimicrobial activity for these compounds. It also reinforces the hypothesis of a global antibacterial action of these molecules that potentially target numerous molecular components of bacterial cells simultaneously^9^. Triclosan is the only biocide included in our study that displayed a strong concentration-dependent activity. Noteworthy, it is also the only biocide of our panel for which a molecular target is known. Triclosan inhibits lipid synthesis by targeting the enoyl reductase, an essential enzyme involved in the fatty acids elongation cycle, demonstrated in *Escherichia coli* (FabI enzyme) and in *Mycobacterium smegmatis* (InhA enzyme)^11^. We hypothesize that triclosan acts similarly in both *S. aureus* and *E. faecium* as an antibiotic analog by binding to specific molecular target(s) in agreement with its antibacterial effect that is strongly concentration-dependent.

In this report, we investigated, for the first time, if biocides could influence sRNA expression in two bacterial pathogens, reasoning that biocide are environmental stresses that could be sensed and detected by sRNA-associated gene regulatory networks, to cope with the trigger. sRNA expression study on both *S. aureus* (3 sRNAs), and *E. faecium* (9 sRNAs) in the presence/absence of biocide SIC, revealed that expression levels of several sRNAs is strongly reduced as a result of biocide SIC exposure, although the roles of and the mechanisms connecting those RNAs with the response of bacteria to biocide is unknown. The sRNAs from *S. aureus* were selected among dozens of expressed sRNA in this bacterium (http://srd.genouest.org/)^33^ because of their implications in either bacterial virulence (SprD , RNAIII) or antibiotic resistance (SprX)^24–26^. Under biocides conditions, RNAIII and SprX were significantly under- and over-expressed depending of the growth stages. Owing to the large number of regulatory functions of RNAIII^24^, its under-expression under chlorhexidine exposure could globally alter the quorum sensing network since RNAIII is the effector. SprX transcript levels followed a similar kinetic under benzalkonium chloride and PVP-iodine, with initial over-expression before returning to normal at the stationary phase. Thus, SprX is implicated into the response towards several antimicrobials targeting cell wall, such as glycopeptides^25^, benzalkonium chloride (this study) or intracellular components as PVP-iodine (this study). The biological functions of sRNAs recently described as expressed in *E. faecium*^29^ are still under investigations but they could possibly be implicated in gene regulatory networks after a biocide stress is applied and sensed. Among the nine studied sRNAs, sRNA_0160 is a leading candidate due to its substantial repression under chlorhexidine digluconate, benzalkonium chloride, and mostly under triclosan. Furthermore, a previous study showed that sRNA_0160 expression level was significantly downregulated under SIC exposure of daptomycin, an antibiotic targeting the cell wall^29^. Taken together, it suggests that this sRNA would be connected to both the antibiotic and biocide responses in *E. faecium*, and a deeper understanding of its mechanisms of action would benefit to both fields*.*

Based on these results, we noticed that sRNA-mediated responses after exposure to four different biocides are distinct between *E. faecium* and *S. aureus*. Indeed, triclosan exposure did not lead to significant alteration of sRNAs expression in *S. aureus*, although it was related to an overall repression of sRNAs expression in *E. faecium*. By contrast, PVP-iodine modified SprX expression in *S. aureus*, but did not influence sRNA expression levels in *E. faecium*. These results show that biocide exposure induces different sRNA-mediated responses for optimal fitness between the bacterial species. Noteworthy, part of the difference in the transcriptomic response observed between the two strains could be related to their different genetic backgrounds. Indeed, *E. faecium* Aus0004 is a multi-drug resistant isolate^34^ whereas *S. aureus* HG003 is a methicillin-susceptible strain^35^ so that we could hypothesize that the adaptation steps necessary for *E. faecium* to become resistant to antimicrobials could lead to a cross- or a co-resistance selection to biocides. In addition, the expression of a given sRNA can be unchanged, repressed or increased depending on which biocide is used. Furthermore, we should extend our investigations in other bacterial pathogens that may reveal specific variations into their overall sRNA contents under biocides sub-lethal conditions. Likewise, further investigations regarding the functions, mechanisms and molecular targets of these biocide-responding sRNAs are needed to a better understanding of what appear to be complex sRNA-driven regulatory networks allowing biocide adaptation of bacterial pathogens to the presence of biocides into the environment.

## Methods

### Bacterial strains

The *E. faecium* Aus0004 reference strain, isolated from the bloodstream of a patient in Melbourne in 1998, was selected^34^. It is a vancomycin-resistant (*vanB*) strain belonging to clade A1 that is widely prevalent in healthcare facilities across the world^36^. Its genome was sequenced and annotated in 2003 (Genbank accession no. CP003351). The reference strain of *S. aureus* was HG003, described as a model strain for physiology and mechanistic studies^37^. It is derived from *S. aureus* NCTC 8325 (Genbank accession no. NC_007795) that was isolated in 1960 from a septic patient and sequenced in 2001^35^.

### Biocides

Four clinically-relevant biocides were studied: chlorhexidine digluconate, benzalkonium chloride, PVP-iodine and triclosan (Sigma-Aldrich Corp., St. Louis, MO, USA). Chlorhexidine digluconate, benzalkonium chloride and PVP-iodine stock solutions (4096 mg/L) were prepared in sterile water and stocked at 4 ± 2 °C until use. Triclosan powder, poorly soluble in water, was diluted in ethanol (EtOH) 60% solvent and freshly prepared at each assay. The suitability of EtOH 60% solvent was confirmed by innocuity testing (data not shown). Dilutions were prepared from stock solutions in culture media adapted to the experiments: cation-adjusted Mueller–Hinton broth (CA-MHB) (Sigma-Aldrich Corp., St. Louis, MO, USA) for MICs and MBCs assays, brain–heart infusion (BHI) broth (Sigma-Aldrich Corp., St. Louis, MO, USA) for sub-lethal conditions assays, and bacterial cultures for RNA isolation.

### Antimicrobial activity testing

MICs were determined by the broth microdilution (BMD) method in CA-MHB medium after 24 and 48 h incubation at 35 ± 2 °C. Standardized inocula and step-dilutions of biocides were derived from EUCAST recommendations for antibiotic susceptibility testing^38^. MBCs were determined by sub-culturing on Mueller–Hinton agar plates the wells of MIC plate exhibiting bacterial growth inhibition after 24 h of incubation and were defined as the lowest concentration of biocide that reduces the initial bacterial inoculum by ≥ 99.9% (i.e., − 3 Log_10_). Each assay was performed at least in triplicate.

For biocide stress characterization, bacterial strains were exposed to sub-lethal concentrations of biocides. No bacterial growth was observed in microplates in CA-MHB medium therefore, the experiment was performed in BHI medium. Standardized bacterial suspensions in BHI medium were exposed to a biocide gradient (from 1× to 1/128× MIC) at 37 °C without agitation for 12 h. Optical density at 600 nm (OD_600_) was measured at 30-min intervals using a Synergy 2 multi-mode reader (Biotek). The reliability between OD variations and bacterial growth has been assessed by a positive correlation between OD values and CFU/mL in BHI medium without biocide (R_Pearson_^2^ > 0.9) and by the absence of interference of biocide in BHI medium on OD values. Each assay was performed at least in triplicate. Growth curves were represented with the GraphPad Prism software v5.0 (San Diego, CA, USA). Sub-inhibitory concentrations (SIC) corresponded to the highest concentration of biocide for which the standard deviation of the growth curve overlapped with the one of the biocide-free control.

### Bacterial Cultures, RNA Isolation, and Expression Analysis

*Enterococcus faecium* and *Staphylococcus aureus* strains were grown in BHI medium and then harvested at three different growth stages (mid-exponential [ME], late exponential [LE] and early stationary [ES]) that were determined at 2:15, 3:30 and 4:45 of growth time respectively. Cells were isolated by centrifugation and dissolved in a solution of 33 mM sodium acetate, 17 mM sodium dodecyl sulfate, and 1 mM EDTA (pH 5.5). The cells were then mixed with glass beads and lysed by using a Fast Prep instrument (MP Biochemicals, LLC, Santa Ana, CA, USA).

Total RNAs were isolated by using water-saturated phenol (pH 5.0). RNAs were precipitated and washed with ethanol. Depletion of residual DNA was performed using the TURBO DNA-*free* kit (Invitrogen™, Thermo Fisher Scientific). Three independent experiments were performed, with independent RNA purifications.

Expression levels of regulatory RNAs in strains were monitored by using a 2-step quantitative reverse-transcription PCR. Briefly, cDNAs were produced by using a High-Capacity cDNA Reverse Transcription Kit (Applied Biosystems) and amplified by using SYBR Green (Power SYBR™ Green PCR Master Mix, Thermo Fisher Scientific) and specific primers, as previously described^25,26,29^. Regulatory RNA counts were normalized against house-keeping genes (*tmRNA* for *S. aureus*, and *adk* for *E. faecium*) using the comparative cycle threshold ΔΔCt method^39^. Statistical t-test was performed to analyse the ∆Ct under biocide condition compared to the control condition, as described^40^. Fold-differences calculated using the ΔΔCT method were expressed as a range, with incorporation of the standard deviation of the ΔΔCT value into the fold-difference calculation.

## Supplementary Information

Supplementary Information
