# Supplementary material for: Distinct expression profiles of regulatory RNAs in the response to biocides in Staphylococcus aureus and Enterococcus faecium
Source: Sci Rep. 2021 Mar 25;11:6892. doi: 10.1038/s41598-021-86376-y (PMC7994832; doi:10.1038/s41598-021-86376-y)
Supplement: Supplementary file 1 — Supplementary Information [file 41598_2021_86376_MOESM1_ESM.docx]

**Distinct expression profiles of regulatory RNAs in the response to biocides in *Staphylococcus aureus* and *Enterococcus faecium***

Loren DEJOIES^1,2^, Killian LE NEINDRE^1,2^, Sophie REISSIER^2^, Brice FELDEN^2*^, Vincent CATTOIR^1,2,3*^

*^1^Rennes University Hospital, Department of Clinical Microbiology, Rennes, France.*

*^2^University of Rennes 1, Inserm UMR_S 1230, Bacterial Regulatory RNAs and Medicine, Rennes, France.*

*^3^National Reference Center for Antimicrobial Resistance (lab ‘Enterococci’), Rennes, France.*

**Table S1.** Transcript level variations of 9 regulatory RNAs from *E. faecium* under biocide sub-inhibitory concentrations at three growth stages: mid-exponential, late exponential and early stationary. Significant fold differences are presented in bold characters.

| **Regulatory sRNA** | **Fold difference in sRNA relative to untreated** | | | |
| --- | --- | --- | --- | --- |
|  | **Chlorhexidine** | **Benzalkonium chloride** | **PVP-iodine** | **Triclosan** |
| **Mid-exponential** | | | | |
| sRNA_0030 | -2.4  (-2.5 ;-2.2) | -2.2  (-3.1 ; -1.5) | -1.1  (-1.2 ; 1.1) | -1.8  (-2.4 ; -1.4) |
| sRNA_0120 | -1.9  (-2.3 ; -1.5) | -1.7  (-2.2 ; -1.4) | -2.5  (-16.0 ; 2.6) | -1.1  (-2.0 ; 1.6) |
| sRNA_0160 | -2.6  (-3.4 ; -2.0) | 2.5  (1.8 ; 3.5) | -1.1  (-1.6 ; 1.3) | **151.1**  (92.6 ; 246.6) |
| sRNA_0280 | -1.6  (-2.1 ; -1.2) | -3.2  (-4.2 ; -2.4) | -1.7  (-2.1 ; -1.4) | 3.7  (2.2 ; 6.2) |
| sRNA_1260 | -2.3  (-3.3 ; -1.7) | -2.2  (-4.6 ; -1.0) | -2.0  (-3.2 ; -1.2) | -3.1  (-6.1 ; -1.6) |
| sRNA_1300 | -2.0  (-3.7 ; -1.1) | -3.8  (-10.3 ; -1.4) | -1.0  (-1.8 ; 1.7) | 3.0  (1.9 ; 4.8) |
| sRNA_2050 | -1.9  (-4.1 ; 1.1) | -2.0  (-6.4 ; 1.6) | -2.5  (-5.1 ; -1.3) | -1.0  (-1.7 ; 1.7) |
| sRNA_2210 | -1.4  (-1.9 ; -1.1) | -0.9  (-1.0 ; 1.1) | -1.3  (-1.7 ; -1.0) | -1.5  (-2.8 ; 1.3) |
| sRNA_2410 | -1.3  (-1.5 ; -1.2) | -1.2  (-1.8 ; 1.3) | -1.3  (-3.9 ; 2.5) | -2.1  (-3.0 ; -1.5) |
| **Late exponential** | | | | |
| sRNA_0030 | -5.6  (-10.5; -3.0) | -6.7  (-7.8 ; -5.8) | 1.8  (1.3 ; 2.6) | **-17.3**  (-22.2 ; -13.5) |
| sRNA_0120 | **-31.8**  (-37.1; -27.3) | **-43.5**  (-67.8 ; -27.9) | -1.8  (-4.1 ; 1.2) | **-56.7**  (-80.6 ; -39.9) |
| sRNA_0160 | **-81.8**  (-130.0; -51.5) | **-55.1**  (-87.1 ; -34.8) | -1.5  (-3.1 ; 1.3) | **-83.3**  (-118.7 ; -58.5) |
| sRNA_0280 | **-13.4**  (-17.6 ; -10.2) | **-24.6**  (-32.1 ; -18.9) | -1.2  (-1.4 ; -1.0) | **-18.2**  (-33.2 ; -10.0) |
| sRNA_1260 | -3.4  (-9.7 ; -1.2) | -4.4  (-8.5 ; -2.3) | 1.8  (1.6 ; 2.0) | -5.7  (-11.3 ; -2.9) |
| sRNA_1300 | **-12.3**  (-24.2 ; -6.3) | -8.0  (-11.7 ; -5.5) | 2.8  (1.8 ; 4.5) | -6.2  (-9.1 ; -4.2) |
| sRNA_2050 | -2.3  (-5.6 ; 1.1) | -4.5  (-7.3 ; -2.8) | 1.8  (1.3 ; 2.3) | -9.6  (-26.9 ; -3.4) |
| sRNA_2210 | **-15.9**  (-33.9 ; -7.5) | **-25.7**  (-33.5 ; -19.7) | 1.0  (1.0; 1.0) | **-28.6**  (-37.6 ; -21.7) |
| sRNA_2410 | -4.1  (-8.0 ; -2.1) | -4.1  (-4.7 ; -3.5) | -1.5  (-2.7 ; 1.3) | -8.8  (-15.2 ; -5.1) |
| **Early stationary** | | | | |
| sRNA_0030 | **-11.1**  (-15.8 ; -7.8) | **-35.5**  (-36.2 ; -34.8) | -4.2  (-5.7 ; -3.1) | **-69.4**  (-90.7 ;-53.1) |
| sRNA_0120 | **-13.5**  (-24.1 ; -7.6) | **-23.6**  (-50.8 ; -10.9) | -5.4  (-7.9 ; -3.7) | **-145.2**  (-184.2 ; -114.4) |
| sRNA_0160 | **-271.0**  (-287.6 ; -255.3) | **-42.1**  (-50.2 ; -35.4) | -3.7  (-5.9 ; -2.4) | **-45,175**  (-57,158 ; -35,705) |
| sRNA_0280 | **-122.4**  (-268.2 ; -55.9) | **-46.6**  (-61.5 ; -35.3) | -6.2  (-7.6 ; -5.0) | **-1009**  (-1,473 ; -690.6) |
| sRNA_1260 | -2.1  (-4.5 ; -1.0) | **-22.5**  (-23.0 ; -22.0) | -5.9  (-8.0 ; -4.4) | **-20.4**  (-27.7 ; -15.1) |
| sRNA_1300 | -4.9  (-9.3 ; -2.6) | -2.3  (-6.0 ; 1.1) | -2.8  (-4.6 ; -1.7) | **-26.4**  (-48.7 ; -14.3) |
| sRNA_2050 | -3.8  (-5.5 ; -2.6) | **-17.6**  (-23.4 ; -13.3) | -5.8  (-6.9 ; -4.9) | **-13.0**  (-20.4 ; -8.3) |
| sRNA_2210 | -5.6  (-8.2 ; -3.8) | **-19.2**  (-37.8 ; -9.8) | -7.2  (-8.9 ; -5.8) | **-73.9**  (-99.5 ; -54.8) |
| sRNA_2410 | -6.2  (-13.0 ; -2.9) | **-11.5**  (-15.6 ; -8.5) | -5.1  (-6.7 ; -3.8) | **-14.1**  (-19.6 ; -10.2) |

**Table S2.** Expression variations of 3 regulatory RNAs from *S. aureus* under biocide sub-inhibitory concentrations at three growth stages: mid-exponential, late exponential and early stationary. Significant fold differences (fold change ≤-10 or ≥10) are presented in bold characters.

| Regulatory sRNA | **Fold difference in sRNA relative to untreated** | | | |
| --- | --- | --- | --- | --- |
|  | **Chlorhexidine** | **Benzalkonium chloride** | **PVP-iodine** | **Triclosan** |
| **Mid-exponential** | | | | |
| ARN III | **-26.8**  (-191.1; -3.7) | -1.3  (-1.8 ; 1.0) | 2.3  (1.5 ; 3.4) | 1.7  (1.1; 2.6) |
| SprD | -1.3  (-4.3 ; 2.4) | **18.2**  (9.4 ; 35.4) | **14.7**  (11.4 ; 18.8) | -1.8  (-3.0 ; -1.1) |
| SprX | 1.6  (-2.0 ; 5.2) | **18.6**  (8.8 ; 39.2) | **77.6**  (48.3 ; 124.7) | 1.2  (-1.3 ; 1.9) |
| **Late exponential** | | | | |
| ARN III | **-10.6**  (-208.8; 1.9) | -6.2  (-26.8 ; -1.4) | 1.9  (1.3 ; 2.9) | -1.5  (-2.3 ; -1.0) |
| SprD | -1.3  (-3.6 ; 2.0) | 2.0  (1.0 ; 4.2) | 4.1  (3.7 ; 4.6) | -5.0  (-6.0 ; -4.2) |
| SprX | 6.7  (-1.6 ; 69.6) | **57.9**  (40.9 ; 81.8) | **66.2**  (65.0 ; 67.4) | 2.3  (1.6 ; 3.2) |
| **Early stationary** | | | | |
| ARN III | **-1,310**  (-1,365.7 ; -1,256.7) | -4.4  (-6.0 ; -3.2) | 2.3  (1.2 ; 4.4) | 2.4  (2.2 ; 2.5) |
| SprD | -1.7  (-17.9 ; 6.3) | -5.3  (-10.9 ; -2.5) | 1.1  (-1.7 ; 1.9) | 1.3  (1.1 ; 1.7) |
| SprX | 2.3  (-4.3 ; 22.2) | 1.6  (-1.0 ; 2.5) | 4.9  (3.0 ; 7.8) | 3.3  (2.9 ; 3.7) |
